# Recognizing Depression from the Microbiota–Gut–Brain Axis

**DOI:** 10.3390/ijms19061592

**Published:** 2018-05-29

**Authors:** Shan Liang, Xiaoli Wu, Xu Hu, Tao Wang, Feng Jin

**Affiliations:** 1 Key Laboratory of Mental Health, Institute of Psychology, Chinese Academy of Sciences, Beijing 100101, China; liangshan223l@hotmail.com (S.L.); wuxl@psych.ac.cn (X.W.); hux@psych.ac.cn (X.H.); wangt@psych.ac.cn (T.W.); 2 Department of Psychology, University of Chinese Academy of Sciences, Beijing 100049, China

**Keywords:** major depressive disorder, brain–gut axis, microbiota–gut–brain axis, gut microbiota, psychobiotics

## Abstract

Major depression is one of the leading causes of disability, morbidity, and mortality worldwide. The brain–gut axis functions are disturbed, revealed by a dysfunction of the brain, immune system, endocrine system, and gut. Traditional depression treatments all target the brain, with different drugs and/or psychotherapy. Unfortunately, most of the patients have never received any treatment. Studies indicate that gut microbiota could be a direct cause for the disorder. Abnormal microbiota and the microbiota–gut–brain dysfunction may cause mental disorders, while correcting these disturbance could alleviate depression. Nowadays, the gut microbiota modulation has become a hot topic in treatment research of mental disorders. Depression is closely related with the health condition of the brain–gut axis, and maintaining/restoring the normal condition of gut microbiota helps in the prevention/therapy of mental disorders.

## 1. Introduction

Major depressive disorder is one of the leading causes of disability, morbidity, and mortality worldwide. The disorder has already affected over 350,000,000 people, and every one in five people probably suffers from it in one stage of their lifespan [1,2,3]. More than 85% of patients with a first episode will relapse in the next 10 years, and most of the patients have suicidal thoughts, 15–20% of whom will die from suicide [4,5]. As shown in a Nature report in 2014, the years lived with disability (YDLs) brought by depression account for 10.3%, which is more than any other disease [6,7]. The latest report in Lancet in 2017 also showed that the disability-adjusted life years (DALYs) brought by depression had increased between 1990 and 2016 [8].

According to modern psychology and biology conceptions, major depression is not just a mental disorder but also a physiological disease. It has obvious biological foundations, such as brain changes including unbalanced neurotransmitters, impaired neurogenesis, neuroplasticity decline, and abnormal neuronal circuitry [9,10]. Large genome-wide association studies estimated the chances of heritability to be 37% to 48% for major depression [11,12]. However, the ever-increasing incidence of depression deviates from the Hardy–Weinberg Equilibrium, indicating that environmental factors play an important role in the disorder.

It is generally agreed upon that depression is induced by the cumulative effect of genetic information and environmental stresses [11]. Certain genes and psychological features might predispose some people to depression and stressful life events including early-life stress are important inducements of depression [1,13], but the latest research has indicated that gut microbiota probably plays a crucial part in the pathophysiology of depression [14,15,16,17,18].

## 2. The Pathophysiology of Major Depressive Disorder

The pathophysiology of major depression has been increasing clearly following the development of neuroscience and bioinformatics. It mainly involves four aspects, the dysfunction of the brain, the hypothalamus–pituitary–adrenal (HPA) axis, the immune system, and the gut–brain axis. The brain abnormalities are mainly reflected in the unbalanced neurotransmitters, the impaired neuroplasticity, and the abnormal neural circuitry [1,10]. The HPA axis dysfunction is mainly manifested as a maladjustment of negative feedback mechanisms [19,20]. The immune changes are mainly seen as chronic inflammation [21,22]. The gut–brain dysfunction mainly includes gastrointestinal disorders and gut microbiota abnormalities [1,11,23,24,25].

### 2.1. The Brain Dysfunction

Neurotransmitters play a crucial part in the brain and behavior. Depression is inseparable from neurotransmitter imbalance [26,27]. The monoaminergic neurotransmitter deficiency hypothesis posits that positive moods including happiness go hand in hand with monoamine neurotransmitters serotonin (5-HT), norepinephrine (NE), and/or dopamine (DA) and that symptoms of depression arise from insufficient levels of these neurotransmitters. Recovering these neurotransmitter levels will have antidepressive effects [26,28]. However, most of the selective serotonin reuptake inhibitors (SSRIs) work slowly and just bring alleviations for part of the patients, indicating that there are still other mechanisms involved in depression [9]. Subsequent research revealed that also signaling by other neurotransmitters probably changed in depression. For example, the glutamatergic system and acetylcholine system are hyperactive, while the gamma-aminobutyric acid (GABA) system is inhibited [27,29,30].

The prefrontal cortex, hippocampus, and amygdala play a vital role in the regulation of emotion, stress responses, self-control, motivation, and cognitive reaction, but in depressed patients the function of the prefrontal cortex and hippocampus are impaired, while the activity of the amygdala is increased [31]. The traditional brain-derived neurotrophic factor (BDNF) hypothesis posits that BDNF is an important regulator of neurogenesis and that depressive symptoms arise from the decrease in BDNF content and the following increase in neural apoptosis. Therefore, long-lasting antidepressant therapies increase the neurotrophic factors including BDNF, stimulate neurogenesis, reduce the hippocampus neuronal apoptosis, and improve the mood and cognition [1,20,32]. Later research found that depressed patients not only present impaired neurogenesis but also present disturbed neuralgias growth, reduced synaptic plasticity, impaired myelin function, and a decrease in total neuroplasticity [9,10,31]. The new neuroplasticity hypothesis posits that depressive symptoms arise from the impaired neuroplasticity, which can be induced by many risks factors, including neurotransmitter imbalance and insufficient BDNF. Antidepressant therapies focusing on neurotransmitter recovery and brain stimulation work through the increase of neuroplasticity and the decrease of neuronal apoptosis [2,33,34,35]. These theories put emphasis on the changes at the molecular and cellular level, while some other theories, including the neural circuit hypothesis point the changes in function. According to the neural circuit hypothesis, depression occurs as a result of aberrations in communication between specific neural structures of the brain, such as the DA neurons in the ventral tegmental area (VTA) and their projections and 5-HT neurons in the dorsal raphe nucleus and their projections. These abnormalities in neural circuits can be restored via therapies including deep brain stimulation [10,36].

### 2.2. The HPA Axis Dysfunction

The HPA axis is an important part of the stress response system, and dysfunction of the HPA axis is one of the most important mechanisms behind depression [11,21,37,38]. Both psychological and physiological stress activate the HPA axis and stimulate the release of corticotrophin-releasing factor (CRF) and vasopressin (AVP) by the hypothalamus. Both CRF and AVP induce the anterior pituitary gland to secrete adrenocorticotrophic hormone (ACTH), which enhances the release of adrenocortical hormones, including glucocorticoid (GC), and causes an increase in circulatory GC levels, which inhibits the secretion of CRF and AVP by the hypothalamus, forming a negative feedback circuit [37,39]. However, over half of the depressed patients present negative feedback dysfunction of the HPA axis, including a chronic increase in circulatory GC and ACTH, and some of the patients even suffer from hypercortisolemia [21,37]. One hypothesis posits that the glucocorticoid receptor (GR) plays an important role in the HPA axis function during depression; the excessive circulating GC reduces the sensitivity of GR, while antidepressant therapies increase the GR expression, enhance the GR function, and improve the negative feedback medicated by GR [37,38]. Later research found that HPA axis dysfunction also reduces BDNF expression [39], inhibits 5-HT synthesis [40], decreases Glu receptor expression [41], and even disturbs neuroplasticity and neural circuits [10,42].

### 2.3. Immune System Abnormalities

Inflammation is also an important pathological feature of depression. A subpopulation of depressed patients present immune dysregulation and chronic inflammation [21,43,44]. The cytokine hypothesis posits that in depression the proinflammatory cytokines, including IL-6 and TNF-α, increase in amount while the anti-inflammatory cytokines, including interleukin-10 (IL-10) and transforming growth factor-beta (TGF-β), decrease, making the holistic immune response tend to inflammation. The excessive proinflammatory cytokines inhibit the negative feedback of the HPA axis, increase the permeability of the blood–brain barrier, reduce the synthesis of 5-HT, disturb the glutamatergic systems, and result in depression [40,43,45,46,47,48]. The early theories mainly focus on peripheral inflammation, but new theories, such as the neuroinflammation hypothesis and inflammasome hypothesis pay more attention to central inflammation [42,47]. The neuroinflammation hypothesis emphasizes the adverse effects on the central nervous system (CNS) exerted by excessive proinflammatory cytokines released by microglia, which can be induced by various factors such as psychological stress, disease, and infection [42]. The inflammasome hypothesis puts emphasis on the influences of neuroinflammation induced by the inflammasome [49,50]. Neuroglia cells play a vital part in the regulation of neuro immune and neuroplasticity. Although different theories focus on different aspects, all of them hypothesize that the neuroinflammation and neuroplasticity impairment induced by neuroglia dysfunction results in depression [42]. The anti-inflammatory effect of traditional antidepressant therapies is not obvious [44], and combining antiinflammation methods with antidepressive therapies will probably give better results [47].

### 2.4. Gut Brain Dysfunction

The gut of mammals is also called gut brain because it has its own nervous system (enteric nervous system) and can make relatively independent responses to external signals [18,51]. The gut brain is a microbial organ, 90–95% of the total cells of which are microorganisms including bacteria, archaea, fungi, viruses, and some protozoa, and the metabolism, immune system, and signal transmission are all closely related with microbiota. Thus, the gut and gut microbiota can serve as a whole to respond to and influence other organs [52,53,54,55]. Depressed patients often have gut brain dysfunction, such as appetite disturbances, metabolic disturbances, functional gastrointestinal disorders, and gut microbiota abnormalities [15,23,25,56,57,58].

**Figure 1:**
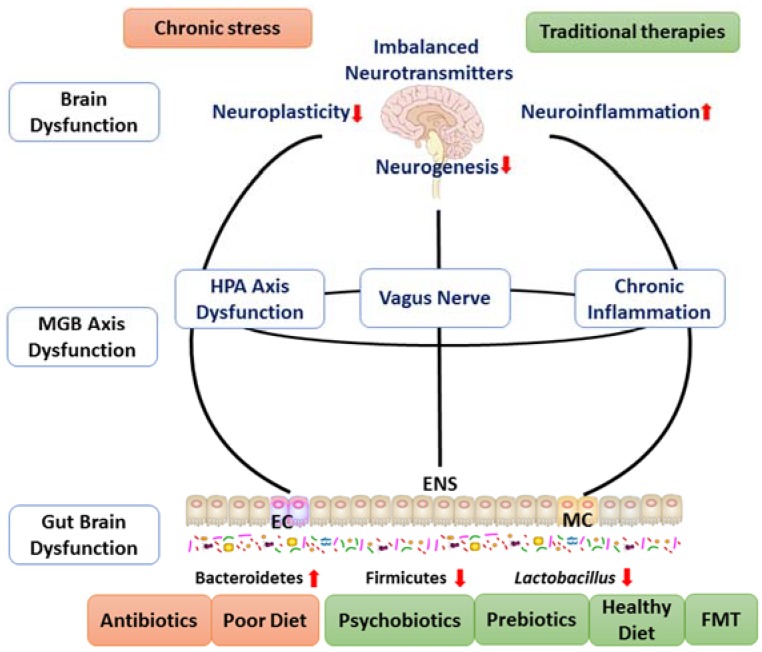
The main pathophysiology and therapy targets of depression. Microbiota–gut–brain axis dysfunction is the main pathophysiology and potential treatment target of major depression. It includes brain dysfunction and gut brain dysfunction. The light red frames in the top and bottom are risk factors of depression, while the light green frames are therapies of depression. EC, enteroendocrine cell; ENS, enteric nervous system; MC, mast cell.

Major depression is not just a simple mental disorder or brain disease, but also a systemic disease. Patients often suffer from various disorders simultaneously, such as brain dysfunction and periphery dysfunction, such as HPA axis disturbances, immune dysregulation, and gut brain disturbances. These disturbances interplay with each other. For example, chronic stress reduces the 5-HT content in the brain; the synthesis and secretion of 5-HT are also influenced by various factors including the HPA axis, immune system, and gut brain, and in turn the 5-HT content affects the function of these organs [20,40,59]. As shown in Figure 1, taking both central and periphery abnormalities into account will facilitate the research of depression, and brain–gut axis dysfunction will explain the pathological basis better.

The brain-gut axis is the bidirectional message transformation pathway between brain and gut in mammals. It connects the brain and gut through several pathways including nerves, the HPA axis, and the immune system [14,18]. Factors such as psychological stress and disease impairing one or more pathways of the brain–gut axis probably induce brain–gut axis dysfunction and result in depression [25,60,61]. Following the development of gut microbiota, researchers not only focus on the top–down effects of the brain–gut axis (from brain to gut), but they also pay close attention to the down–top influences (from gut to brain) [23,25,62]. The functions of many a system, including metabolism, the immune system, the endocrine system, and the nervous system, are all closely related with the gut brain. Changes of the gut brain such as gut microbiota abnormalities influence the brain and behavior, and brain changes regulate the function and construction of the gut brain. Combining the brain and gut brain will probably become the new tendency of neuroscience, and targeting the gut microbiota will possibly be a promising area for the therapy of mental disorders and neurological diseases [18,63,64,65,66,67,68,69,70,71].

According to the gut microbiota hypothesis, the gut microbiota can influence the brain and behavior through the gut–brain axis, which is also called microbiota–gut–brain axis to emphasize the importance of the microbiota. It plays a crucial part in mental disorders [64,65]. Gut microbiota is a key component of the gut brain. It regulates the common functions and build-up of the gut brain [52,53,54,55], influences the development and maturation of the HPA axis [72,73,74,75], affects the development and function of the immune system [76,77,78], regulates the construction of the blood–brain barrier [79], influences the synthesis and recognition of neurotransmitters [59,72,80], affects neurogenesis [81], the development and function of neuralgias [82,83], and the formation of myelination [84], and impresses the development and function of the brain [66,78,85,86]. Thus, regulating gut microbiota cannot only improve gut brain dysfunction but also alleviate abnormalities in the immune system, HPA axis, and brain. All these results are in line with the gut microbiota hypothesis, which will probably be the promising direction of mental disorder therapy and prevention.

## 3. The Latest Research Progress of Depression: The Microbiota Hypothesis

The gut microbiota hypothesis posits that depression is closely related with gut microbiota, and microbiota–gut–brain axis dysfunction is the main pathological basis of depression. Gut microbiota abnormalities are a direct inducement and key risk factor hiding in environmental and genetic risk factors, and that microbiota regulation is the promising method for depression therapy and prevention. An increasing amount of research, exploring the gut brain in the last decades supports the hypothesis from different aspects [18,65,67,68,87,88,89,90,91,92].

### 3.1. Depressed Patients Have Different Gut Microbiota from Healthy Persons

Clinic studies have presented that the gut microbiota of depressed patients is significantly different from that of healthy controls. Some research found that both the microbiota diversity and richness declined in patients [57,87]. On the phylum level, the richness of Bacteroidetes and Proteobacteria increased while the richness of Firmicutes decreased; on the family level, the relative abundance of *Prevotellaceae* increased; on the genus level, the abundance of *Prevotella* increased, while the abundance of *Faecalibacterium* and *Ruminococcus* decreased [57,93]. The abundance of *Lactobacillus* and *Bifidobacterium* also declined [94]. Although all these studies have shown the gut microbiota abnormalities of depressed patients, the definite distinctions between that of patients and controls are still in debate [58,95,96], which is probably correlated with the differences of diagnostic criteria, grouping criteria, detection methods of fecal microbiota, etc.

Animal studies also presented the microbiota differences between depressive model animals and control animals. A variety of depression models have shown the phenomenon, including the bilateral olfactory bulbectomy model [97], maternal separation model [98], social disruption model [99], chronic variable stress model [100], and our chronic restraint stress model [101]. Furthermore, the microbiota of depressed animals have similarities with those of depressive patients; for example, the richness of Bacteroidetes increases while the richness of Firmicutes decreases and the abundance of *Lactobacillus* declines [97,98,100].

All of these investigations have suggested that depression is probably linked with certain gut microbiota phenotypes.

### 3.2. Depressive Symptoms Can Be Transmitted Following Fecal Microbiota Transplantation

The mental flu depression seems to have the same infectivity as flu to be transmitted from one subject to other, but the transmission medium, the gut microbiota of depressive patients, is more complex, and it is impossible to transfer under natural conditions.

After transplanting the fecal microbiota of depressed patients and healthy persons to germ-free mice, Zheng et al. found that the depressed recipient mice presented more depressive symptoms, and their microbiota were different from those of healthy recipients. These differences were similar to the differences between their respective human donors, indicating certain microbiota phenotypes can induce depressive symptoms through metabolism changes [95]. Kelly et al. studied another model, in which they transplanted the fecal microbiota of depressed patients to rats which were microbiota-depleted via antibiotic cocktail treatment. The recipient rats of patients presented obvious depressive symptoms, such as anhedonia, an increase in anxiety-like behavior, and tryptophan metabolism disturbances, all of which were similar to those of their microbiota providers [87]. Both the above studies showed that the psychological and physiological symptoms of depression can be transferred between different subjects, further indicating that psychological states are regulated by gut microbiota.

Offspring acquire similar microbiota from parents via longitudinal and horizontal gene transfer under natural conditions [102]. Our unpublished data also imply that the heredity of depression partly lies on the susceptible microbiota obtained from parents.

### 3.3. Gut Microbiota Disturbances Increase the Susceptibilities of Depression

Antibiotics damage the microbiota and increase the incidence of depression. Although antibiotics have played a vital role in human anti-infective therapy, they not only kill pathogens but also destroy beneficial microorganisms, induce microbiota–gut–brain axis dysfunction, and increase the incidence of various diseases, including mental disorders [103,104,105,106]. Large-scale human studies have revealed that the use of antibiotics in antiinfection therapy significantly increases the risk of mental disorders such as depression. The risk presents dose-dependent and time-dependent effects, which means the risk is positively correlated with the dose and time of antibiotic use; this elevated risk still exists 10 years after antibiotic use [107,108]. Study on infants also showed that infants who experienced antibiotic treatment during the first year of their lives had more possibilities to suffer from behavioral problems and depression, and the effects were obvious at the age of three [109]. Animal studies presented a similar phenomenon [104,110].

Stress disturbs the microbiota and increases the susceptibility of depression. Stressful life events are important inducements of depressive disorders, and they are often used in animal depression research. Chronic stress not only impacts the mind and the stress response system, but also disturbs the gut microbiota [111,112,113,114,115,116,117]. Our previous study also showed that chronic restraint stress disturbed the gut microbiota, inducing microbiota–gut–brain axis dysfunction including decreased hippocampus 5-HT content, reduced BDNF mRNA expression, increased plasma stress hormone levels, declined circulatory IL-10 levels, and abnormal gut microbiota, resulting in depression [101].

Diet is one of the most influential factors on the gut microbiota after weaning, and poor diets significantly perturb microbiota and increase the incidence of depression [102,118,119,120]. Many unhealthy diets, including the Western diet, the refined-food diet, and industrially processed food, which contains excessive saturated fat, sugar, and food additives, destroy normal gut microbiota and increase the susceptibility of depression [121,122,123]. The influences of poor diet are probably closely related with the dysfunction of the microbiota–gut–brain axis it induces [124,125].

Antibiotics, chronic stress, and poor diet all disturb gut microbiota, change the microbiota towards a depression phenotype, and increase the incidence of depression. These three factors often appear simultaneously but are ignored by the human host [126,127].

### 3.4. Gut Microbiota Restoration Alleviates Depression

Gut microbiota are closely linked with host health and disease. Microbiota dysbiosis can induce various physiological and psychological diseases, and microbiota restoration brings improvement to these diseases [65,66,128,129,130]. There are four main effective methods to recover normal microbiota, which are probiotics, prebiotics, a healthy diet, and fecal microbiota transplantation (FMT) [120,131,132,133].

Probiotics are defined as “live microorganisms which when administered in adequate amounts confer a health benefit on the host” [134]. Their beneficial effects not only locate in gut, but reach the whole microbiota–gut–brain axis; researchers call these probiotics psychobiotics to emphasize their capabilities to improve behavior and mind [135]. Both clinic and animal research have shown that psychobiotics supplementation alleviates depression symptoms, even achieving similar effects to traditional antidepressant therapies. In double-blind, randomized, placebo-controlled studies, psychobiotics treatments alleviated the depressive and anxious symptoms of patients and improved cognition and metabolism [136,137,138]. Animal studies indicated that the antidepressant effects of psychobiotics are closely related with the regulation of the microbiota–gut–brain axis [99,101,139,140]. The psychobiotics that have been reported mostly belong to lactic acid bacteria, such as special strains of *Lactobacillus casei* [136,139], *Lactobacillus helveticus* [101], and *Bifidobacterium bifidum* [136,139].

Prebiotic is defined as “a substrate that is selectively utilized by host microorganisms conferring a health benefit” [141]. Prebiotics not only regulate gut microbiota but also improve behavior and cognition acting as psychobiotics. These effects are probably achieved through the functional improvement of the microbiota–gut–brain axis [131,142,143,144]. Prebiotics are a hot research topic; and the most popular kinds are fructose-oligosaccharide, galactooligosaccharide, omega-3 fatty acids, etc. [141].

Contrary to poor diets, healthy diets increase gut microbiota diversity and stability and improve health and wellbeing [16,131,145,146]. Healthy diets, including the Mediterranean diet, are rich in dietary fiber, unsaturated fatty acids, and fermented food such as yoghurt, cheese, and natto; they contain less refined carbohydrates, saturated fatty acids, sugar, and food additives. Healthy diets can stimulate the proliferation of beneficial microorganisms and improve behavior and cognition, probably through the microbiota–gut–brain axis [16,120,125,133,145]. These studies have greatly supported the ignored dietotherapy of depression [16].

FMT is the process of transplanting feces from a healthy donor to the receiver’s gut in order to recover the impaired gut microbiota. FMT has played important roles in the treatment of *Clostridium difficile* infection, inflammatory bowel disease, ulcerative colitis, etc. [147]. Some researchers have attempted to treat mental disorders including depression, anxiety, and autism.

According to existing research and applications, recovering gut microbiota via psychobiotics, prebiotics, a healthy diet, and FMT and improving the function of the microbiota–gut–brain axis will play a crucial part in the treatment of mental disorders including depression [68,135,148].

### 3.5. The Mechanisms of Traditional Antidepressant Therapies are Probably Related with Microbiota

It is generally recognized that traditional antidepressant therapies alleviate mental and brain abnormalities and treat depression. But the latest research has found that traditional therapies not only regulate the brain but also influence gut microbiota; their antidepressant effects are probably partly related with the regulation of the microbiota–gut–brain axis [16,149,150].

Medical therapy regulates the gut microbiota. The first antidepressant, isoniazid, was originally used to treat *Mycobacterium tuberculosis* infections; the first-generation tricyclic antidepressants (TCAs) can inhibit the proliferation of many bacteria, including *Escherichia coli*, *Yersinia*, and *Plasmodium*; the currently common SSRI antidepressants can inhibit the proliferation of Gram-positive bacteria; even the recently developed antidepressant ketamine can inhibit the proliferation of *Staphylococcus*, *Enterococcus*, and *Candida albicans*. Common antibiotics, including ceftriaxone sodium and minocycline, present some antidepressant effects [149]. Citalopram showed antidepressant effects in our chronic restraint stress rat model [101]; it also changed the gut microbiota toward a new adaptation, different from the control. This indicates that the effects of antidepressants are probably correlated with gut microbiota. They also remind that considering gut microbiota possibly helps the therapy of treatment resistant depression.

Other therapies also changed the gut microbiota. Except the dietotherapy mentioned above, exercise therapy probably improves depression via the regulation of gut microbiota and the microbiota–gut–brain axis; a poor sedentary life style characterized by a lack of exercises increases the incidence of depression, while getting enough exercise alleviates depression [150,151].

### 3.6. New Therapies Integrating Gut Microbiota Regulation Present Promising Effects

Some researchers have tried to use new antidepressant therapies emphasizing gut microbiota regulation. In 2016, Schnorr and Bachner combined dietotherapy and psychotherapy to treat a panic attack patient; they also removed the foods that caused long-duration blood sugar spikes from and increased the food rich in probiotics in the diet. The integrated treatment alleviated the anxiety and insomnia symptoms of the patient, increased the beneficial bacterial abundances in feces, including *Lactobacillus*, reduced the harmful bacteria, including *Clostridium*, and changed the microbiota composition and diversity [152]. Bambling et al. conducted a pilot study in 2017, in which they used a new therapy combing probiotics, magnesium orotate, and SSRIs for the treatment resistant depression. The depressive symptoms were significantly improved after 8 weeks of intervention; the patients had relapsed after cessation of the test intervention while still on SSRI medication [153]. The above two studies indicate that better effects will be achieved if the gut microbiota is also taken into account in antidepressant therapies.

In conclusion, depressive patients had abnormal gut microbiota, which were probably induced by various factors, including antibiotic use, stress, poor diet, hereditary susceptibility, etc. This depressive gut microbiota phenotype is relatively stable and can be transferred from one subject to another in particular situations. Impairing the normal microbiota increases the incidence of depression, while microbiota recovery using many ways including psychobiotics, prebiotics, FMT, healthy diets, exercises, and medication has antidepressant effects.

## 4. Conclusions and Outlook 

In the past 100 years, scientists have made great progress in depression research, and many antidepressant therapies have been developed, among which the most common is medical treatment [154]. However, currently available treatments are limited by low rates of efficacy, therapeutic time lag, and undesirable side effects [3,10,155]. For a variety of reasons, including stigma, most people with depression go undiagnosed or untreated (approximately 75% in the UK and over 92% in China). Even if the disorder is diagnosed, today’s medications will work well for only about half of those who seek help [3,6,156,157].

Negative cognitive style, unbalanced brain neurotransmitters, declined neuroplasticity, abnormal neural circuits, HPA axis dysfunction, chronic inflammation, and gut brain dysfunction are common symptoms of depression, several of which usually present at the same time, indicating that microbiota–gut–brain axis dysfunction is probably the main pathological mechanism of depression [69,70,71,158,159,160]. The focus of depression research has transferred from the mind to the brain, to other systems, to the brain-gut and gut-brain axis, and finally to the microbiota–gut–brain axis. Gut microbiota abnormalities can directly induce depression, gut microbiota can influence the behavior and mind via the microbiota–gut–brain axis, and microbiota–gut–brain axis dysfunction is the main pathophysiology of depression, according to the gut microbiota hypothesis. According to the hypothesis, gut microbiota regulation and the following microbiota–gut–brain axis improvement will alleviate and treat depression. Many microbiota recovery methods have been established, including supplementation with psychobiotics and/or prebiotics, diet regulation, and FMT.

For a long time, the antidepressant therapies usually targeted the brain abnormalities, while the dysfunction of other organs was ignored or supposed to alleviate following the improvement of brain function. Regulating the gut microbiota and improving the microbiota–gut–brain axis function will probably bring far-reaching influences to the therapy and prevention of depression. The therapy has higher flexibility and operability compared with traditional therapies, and it is easier for patients to accept and easier for high-risk individuals to prevent depression.

More than 100 years ago, Metchnikoff proposed that abnormal gut microbiota were probably the root of mental disorders including depression and anxiety, and supplementation with probiotics improved these disorders, but the theory has been neglected because of various limitations [161,162,163]. Logan and Katzman suggested that probiotics may be an adjuvant therapy for major depression in 2005 [56]. Scientists have started to pay attention to the role of the brain–gut axis in depression since 2009 [14,18,23,62,164,165]. Dinan et al. proposed the concept of “psychobiotics” to emphasize the potential of probiotics in the treatment of mental disorders [135]. Almost at the same time, some probiotics were proved to have antidepressant effects in animal depression research [101,140]. Since then, the clinical research using probiotics to treat depression has been increasing [136,137,138]. Recently, scientists have tried to treat treatment resistant depression using therapy integrating probiotic supplementation and SSRI medication [153]. It is expected that therapies targeting gut microbiota and the microbiota–gut–brain axis will play an important role in the treatment and prevention of depression in the near future.
